# *Spirulina* Extract Enhanced a Protective Effect in Type 1 Diabetes by Anti-Apoptosis and Anti-ROS Production

**DOI:** 10.3390/nu9121363

**Published:** 2017-12-15

**Authors:** Jiyeong Lee, Arum Park, Mi Jeong Kim, Hee-Joung Lim, Young-Ah Rha, Hee-Gyoo Kang

**Affiliations:** 1 Department of Biomedical Laboratory Science, College of Health Sciences, Eulji University, Seongnam 13135, Korea; bjssw@naver.com (J.L.); latias94@naver.com (M.J.K.); 2 Department of Senior Healthcare, BK21 Plus Program, Graduate School, Eulji University, Seongnam 13135, Korea; sksskdi5959@naver.com; 3 Forensic Science R & D Laboratory, Police Science Institute, Chungcheongnam-do 31539, Korea; limhj@police.go.kr; 4 Department of Food Science & Food Technology, Eulji University, Seongnam 13135, Korea

**Keywords:** antioxidant effect, cytokine, cytotoxicity, *Spirulina* extract, type 1 diabetes

## Abstract

Interest in the nutritional value and pharmacological activities of blue-green algae has gradually increased. *Spirulina* extracts show protective effects against apoptosis and inflammatory damage in various cell types. Here, we investigated the protective effects of extracts from *Spirulina maxima* in a cytokine-mediated type 1 diabetes model in vitro and in streptozotocin-induced diabetic Wistar rats in vivo. Interleukin-1β and interferon-gamma induced substantial cytotoxicity to RINm5F rat insulinoma cells, increasing nitric oxide (NO) production, nuclear factor-kappa B (NF-κB) activity, the expression of endoplasmic reticulum (ER) stress genes, and activation of mitogen-activated protein kinases and key genes related apoptosis. However, the cytotoxicity of cytokines was significantly attenuated by *Spirulina* extract, which effectively prevented NO production by inhibiting the synthesis of cytokine-activated NO synthase (iNOS), and apoptosis was suppressed. These results suggest that *Spirulina* extract might be effective to preserve the viability and function of pancreatic β-cells against cytotoxic conditions. Moreover, diabetic mice orally administered *Spirulina* extract showed decreased glucose levels, increased insulin, and improvement in liver enzyme markers. The antioxidant effect of *Spirulina* extract may be helpful in treating type 1 diabetes by enhancing the survival, and reducing or delaying cytokine-mediated β-cells destruction.

## 1. Introduction

*Spirulina maxima* is a planktonic filamentous cyanobacterium with characteristic morphological features such as the arrangement of multicellular cylindrical trichomas in a helix along the entire body length. *S. maxima* mainly inhabits tropical and subtropical waters, forming massive populations with characteristically high levels of carbonate and bicarbonate as well as a high pH (up to 11). *Spirulina* contains an unusually high amount of proteins, all essential amino acids, essential fatty acids, minerals, vitamins, and photosynthetic pigments [1]. *Spirulina* has recently risen to the fore of research in nutrition because of its impressive nutrient composition, which has been shown to be effective in the treatment of several conditions. In particular, pharmacological analyses have demonstrated the beneficial properties of *Spirulina* both in vitro and in vivo, including antioxidant [2], immunomodulation [3,4], antiviral [5], anticancer [5,6], cholesterol reduction [7], and anti-diabetes [8] activities. In addition, *Spirulina* has an antioxidant defense system, which removes reactive oxygen species (ROS) that can damage cells by inducing oxidative stress [9,10]. Because this antioxidant system reduces most oxidized forms by photosynthesis, the antioxidant property in *Spirulina* extract is associated with some phycobiliproteins, such as C-phycocyanin and allophycocyanin [11,12]. Despite these pharmacological benefits, the molecular mechanisms related to the anti-oxidant effects of *Spirulina* are mostly unknown. Therefore, we sought to determine the effects of *Spirulina* extract on cytokine-induced cytotoxicity in diabetes models, as well as the underlying mechanism, in order to establish its potential in the protection against pancreatic β-cell death.

Type 1 diabetes mellitus is an autoimmune disease resulting from the destruction of insulin-secreting β-cells of the pancreas causing a substantial β-cell deficit. During the early stages of type 1 diabetes, histological findings have revealed features of insulitis and local inflammation, which is characterized by the infiltration of activated macrophages, natural killer cells, and T lymphocytes into pancreatic islets [13]. Complex cross-talk among various immune cells and their released cytokines leads to the selective death of β-cells and consequent insulin deficiency. Proinflammatory cytokines such as interleukin (IL)-1β, interferon-gamma (IFN-γ), and tumor necrosis factor-alpha (TNF-α) play major roles in β-cell function and apoptosis [14].

Cytokines modify the expression of several genes regulated by transcription factors (e.g., NF-κB, STAT-1, and IRF-3) in β-cells [14]. When the transcription factor NF-κB is inactive, it resides in the cytoplasm bound to the inhibitor of κB (IκB). In β-cells, IL-1β, alone or in synergy with IFN-γ, induces the proteasomal degradation of IκB to activate NF-κB, which is then translocated to the cell nucleus, where it regulates gene expression [15]. In particular, NF-κB upregulates the expression of IFN-γ-induced protein 10 kDa (IP-10) and IL-15, which can attract mononuclear cells to the site of insulitis [16]. Moreover, NF-κB promotes induced nitric oxide synthase (iNOS) gene expression and the subsequent formation of nitric oxide (NO), which mediates the destruction of β-cells. In turn, NO downregulates the expression of pancreatic and duodenal homeobox 1 (PDX-1) [16], a transcription factor responsible for β-cell differentiation and function, and sarcoendoplasmic reticulum Ca^2+^ ATPase (SERCA) protein, which pumps Ca^2+^ into the endoplasmic reticulum (ER) via NO production [17]. Therefore, this cytokine-induced NF-κB activation plays a critical role in ER Ca^2+^ homeostasis, attracting immune cells, and β-cell differentiation and function to directly contribute to β-cell apoptosis.

Mitogen-activated protein kinases (MAPKs) play an important role in regulating complex cellular responses by responding to various extracellular stimuli such as osmotic stress, mitogens, heat shock, and pro-inflammatory cytokines. In addition, MAPK-mediated oxidative stress signaling has been implicated in cell death [18]. In the insulin-producing cell line RINm5F, IL-1β, IFN-γ, or TNF-α induce C-Jun N-terminal kinase (JNK) activity, resulting in IL-1β-induced apoptosis [14,16], which has also been demonstrated in insulin-secreting cells [19,20,21]. Further, the suppression of cytokine induction using a small-molecule JNK inhibitor could improve cell viability in human islets [22]. Since cytokine induction through MAPKs, ERK, and p38 signaling has a negative effect on β-cells, both ERK and p38 appear to be involved in mediating the effects of IL-1β on iNOS expression and NO production, respectively [23], with consequences for cytokine-induced β-cell apoptosis [24,25]. 

Moreover, oxidative stress, caused by an imbalance in ROS production and the antioxidant system to suppress ROS activity [11,26], is related to pancreatic β-cell damage, which might also be mediated through cytokines. Several studies have identified the deleterious cytokine effects in pancreatic β-cells [27,28], and a negative relationship between ROS levels and antioxidant enzyme levels has been shown to cause oxidative damage to pancreatic β-cells [29,30]. Furthermore, NF-κB is activated by ROS [31,32] and is inhibited by antioxidants [33]. Reactive nitrogen species (RNS) act as NOSs [16], including NO, nitrogen dioxide, and peroxynitrite, and consist of nitrogenous products [34] that work together with ROS to damage cells, causing nitrosative stress. Cytokines stimulate the NF-κB pathway to induce NO by increasing the expression of iNOS [35], and several in vitro studies have found that NO is associated with the cytokine-induced destruction of β-cell secretory function and cell death [36]. Indeed, a deficit of NO in iNOS-deficient mice protects against chemically induced diabetes [37]. Therefore, NO appears to be an important factor regulating both the function and apoptosis of β-cells in type 1 diabetes.

According to this background, we established an in vitro model of cytokine-induced type 1 diabetes and determined whether treatment with *Spirulina* could protect against cytokine-induced apoptosis of β-cells through oxidative stress, and the involvement of NF-κB and the MAPK signaling pathway in the underlying mechanism. We further established a streptozotocin (STZ)-induced diabetic rat model to evaluate the effects of the oral administration of *Spirulina* extract on pathological characteristics, islet cell pathology, and related enzymes.

## 2. Materials and Methods 

### 2.1. Preparation of Spirulina Extract

*S. maxima* strain UTEX LB2342 (included in *Arthrospira maxima*) was purchased from the UTEX Culture Collection of Algae (University of Texas at Austin). For clonal culture, a single trichome obtained by micropipetting was cultured in sterile Society of Toxicology (SOT) medium (16.8 g NaHCO_3_, 0.5 g K_2_HPO_4_, 2.5 g NaNO_3_, 1 g K_2_SO_4_, 1 g NaCl, 0.2 g MgSO_4_Æ_7_H_2_O, 0.04 g CaCl_2_Æ_2_H_2_O, 0.01 g FeSO_4_Æ_7_H_2_O, 0.08 g Na_2_EDTA, 0.03 mg H_3_BO_3_, 0.025 mg MnSO_4_Æ_7_H_2_O, 0.002 mg ZnSO_4_Æ_7_H_2_O, 0.0079 mg CuSO_4_Æ_5_H_2_O, and 0.0021 mg Na_2_MoO_4_Æ_2_H_2_O in 1 L of distilled water). The strain was maintained under aeration with filtered (0.22 mL) ambient air at 32 °C and a 12:12 light:dark cycle. 

The extract was prepared from *S. maxima* using the crude water extract procedure as previously described [38]. Extraction was carried out with distilled water (DW) at 40 °C for 8 h or with ethanol (EtOH) at 70 °C for 1 h, and then the extracts were centrifuged at 3500 rpm for 5 min, the precipitate was discarded, and the supernatant was lyophilized.

### 2.2. Protein Profile of Spirulina Extracts with Sodium Dodecyl Sulfate-Polyacrylamide Gel Electrophoresis (SDS-PAGE) 

To compare the proteins extracted from *Spirulina*, the extracts were subjected to SDS-PAGE and silver-stained for visualization using a protein silver staining kit (GE Healthcare, Little Chalfont, UK) according to the manufacturer instructions. In brief, the samples were mixed with 4X LDS sample buffer (Invitrogen, Carlsbad, CA, USA), boiled for 5 min, and those containing 1 μg of protein were subjected to 12% SDS-PAGE. The gels were washed for 2 h with shaking in a fixing solution, and then for a further 2 h with shaking in a sensitizing solution, followed by a final wash in DW for 15 min five times. The gels were reacted with the silver solution for 2 h and developed with a developing solution until the bands were visible. Development was stopped with a stop solution, and the gels were preserved in an 8.7% glycerol solution. 

### 2.3. Insulinoma Cell Culture and Treatments 

The rat pancreatic β-cell line RINm5F was purchased from the American Type Culture Collection and cultured in a humidified incubator at 37 °C with 5% CO_2_. The cells were grown in RPMI-1640 medium supplemented with 2500 mM glucose, 10 mM HEPES, 18 mM sodium bicarbonate, 1 mM sodium pyruvate (Sigma, St. Louis, MO, USA), 10% fetal bovine serum, 100 U/mL potassium penicillin, and 100 mg/mL streptomycin sulfate (Gibco, Grand Island, NY, USA).

For treatment, lyophilized *Spirulina* extract or C-phycocyanin (PC; Sigma) was dissolved in the culture medium. Lipopolysaccharide of the *Spirulina* extract was removed by incubation with polymixin B (Sigma) as previously described [38]. Cells were co-treated with or without IL-1β (50 U/mL), IFN-γ (100 U/mL) (R&D Systems, Abingdon, UK), *Spirulina* extract (1 μg/mL), and PC (1 μg/mL) to evaluate the protective effects of *Spirulina* extracts. PC is a phycobiliprotein with nutritional and therapeutic value showing antioxidant, anti-inflammatory, neuroprotective, and hepatoprotective effects. Since PC is a known constituent of *Spirulina* [39], it was used to directly compare the effects to those of *Spirulina* extract.

### 2.4. XTT Assay for Cell Viability

The viability of cultured cells was examined using the XTT assay (Roche, Basel, Switzerland) according to the manufacturer’s instructions. In brief, treated RINm5F cells were cultured in 96-well plates (20,000 cells per well) in 100 μL of medium. After 24 h or 48 h, 50 μL of the XTT labeling mixture was added to each well to a final XTT labeling reagent concentration of 0.3 mg/mL with an electron coupling reagent. After incubation at 37 °C for 8 h, the chemiluminescence intensity was assessed at 450 nm using a GENios ELISA reader (Tecan, Chapel Hill, NC, USA).

### 2.5. 5-Bromo-2-Deoxyuridine (BrdU)-Labeling Cell Proliferation Assay

The BrdU enzyme-linked immunoassay (ELISA) kit (Roche) was used to measure the extent of DNA synthesis in proliferating cells. The principle behind this method for detecting cell proliferation is based on incorporation of the pyrimidine analogue BrdU into DNA during cell proliferation instead of thymidine. In brief, treated RINm5F cells were seeded at 20,000 cells per well in 96-well plates and incubated for 4 h after adding 10 μM BrdU. The cells were added to FixDent solution for fixation and incubated for 30 min at room temperature (25 °C). The cells were then incubated with peroxidase-conjugated mouse monoclonal anti-BrdU-POD antibody followed by incubation for 90 min at room temperature, washed with phosphate-buffered saline (PBS) three times, and then treated with peroxidase as a substrate. The chemiluminescence intensity was measured at 450 nm using the GENios ELISA reader.

### 2.6. Immunocytochemistry and Image Analysis

The cells (10,000) were seeded on coverslips, cultured in 4-well plates. Control cells were cultured for 24 h without cytokines and *Spirulina* (Spi) extract or PC. The cells were fixed with paraformaldehyde for 30 min. After washes in PBS, the cells were treated with blocking medium (0.3% Triton X-100, 0.1% bovine serum albumin in PBS) for 45 min at room temperature, incubated with anti-NF-κB (p65 subunit) (Santa Cruz Biotechnology, Santa Cruz, CA, USA), and diluted in 0.1% bovine serum albumin in PBS for 1 h. The cells were then incubated with a 1:1000-diluted fluorescein isothiocyanate-conjugated secondary antibody for 15 min. The cell nuclei were stained with DAPI (1:1000 in PBS) and washed with PBS three times. The stained cells on the slides were mounted by mounting medium, and the images were detected using laser-scanning confocal microscopy (Zeiss LSM 510, Carl Zeiss, Jena, Germany).

For analysis of NF-κB activity, the regions of interest of each cell obtained from the confocal microscope software were drawn around the cell and nucleus as previously described [40]. The ratios of stained cells and nuclei were calculated as follows:

Ratio = (nuclear intensity − background intensity)/(cell intensity − background intensity)

### 2.7. Western Blot Analysis

Western blot analysis was carried out using antibodies targeting NF-κB p65, IκB**α**, iNOS (Santa Cruz Biotechnology), JNK, phospho-JNK, p38, phospho-p38, ERK, and phospho-ERK (Cell Signaling Technology, Beverly, MA, USA). To obtain proteins, the treated cells were centrifuged, washed with PBS three times, and lysed in RIPA lysis buffer including propidium iodide (UpstateBiotechnology, Lake Placid, NY, USA). After removing the cellular debris, the supernatant was obtained and stored at −20 °C until use. The protein concentration in lysates was measured with the Bradford method using standard controls.

Whole cell lysates were mixed with 4X LDS sample buffer (Invitrogen), boiled for 5 min, and the samples containing 40 μg of protein were subjected to 10% SDS-PAGE. The proteins were then transferred to Hybond-ECL nitrocellulose membranes (GE Healthcare). The membranes were blocked with 5% non-fat milk in TBST buffer (50 mM Tris-HCl, pH 7.5, 150 mM NaCl, and 0.1% Tween 20) for 1 h. The membranes were incubated with the primary antibodies (1:2000) diluted in blocking buffer for 1 h at room temperature, and then incubated with the secondary horseradish peroxidase-conjugated antibodies (1:10,000). The target proteins were detected on X-ray film using ECL Advanced Western Blotting Detection Kit (GE Healthcare). GAPDH was used as a loading control.

### 2.8. RNA Isolation and Real-Time Reverse Transcription-Polymerase Chain Reaction (RT-PCR)

Total RNA was extracted from treated cells using Trizol reagent (Invitrogen) in accordance with the manufacturer’s instructions. RNA was added to chloroform and isopropanol to remove Trizol and the precipitate, respectively. The pellets were air-dried and dissolved in DEPC-treated DW. cDNA was obtained using a cDNA synthesis kit (Fermentas, Hanover, MD, USA) for real-time PCR. The real-time PCR mixture was adjusted to a final volume of 20 μL by adding 30 ng reverse-transcribed total RNA, 167 nM forward and reverse primers, and 2X PCR master mixture (Kapa Biosystems, Boston, MA, USA). The primer sequences, product sizes, and conditions are listed in Table 1. All PCRs were performed in triplicate using the ABI Prism 7000 Sequence Detection System (Applied Biosystems, Carlsbad, CA, USA). 

### 2.9. Measurement of NO and ROS/RNS

The RINm5F cells were seeded at 5,000,000 cells per well in 6-well plates in 2 mL complete medium. After the cells were treated with IL-1β, IFN-γ, *Spirulina* extract, and PC for 24 h, the NO level was assessed by measuring the level of accumulated nitrite in the cell culture media after adding Griess reagent (Promega, Madison, WI, USA), which results in a color reaction to pink that was detected by measuring the absorbance at 560 nm on the GENios ELISA reader. The assay was standardized using various dilutions of 0.1 M sodium nitrite. 

ROS/RNS were measured with H_2_DCFDA (Molecular Probes, Carlsbad, CA, USA), which is a cell-permeable dye that is cleaved by esterases in the cell. Through the action of different ROS (hydroxyl radical, peroxyl radical, H_2_O_2_, and superoxide anion) and RNS (peroxynitrite anion and NO), H_2_DCF is oxidized to its fluorescent form. Therefore, the fluorescence emitted is proportional to the ROS/RNS accumulated in the cells. RINm5F cells (1,000,000) were plated on 6-well culture plates and treated with IL-1β, IFN-γ, *Spirulina* extract, and PC for 24 h. The treated cells were then added to a DCFH-DA solution at a final concentration of 10 μM and incubated at 37 °C for 30 min. The DCF intensity of the labeled cells was measured on the GENios ELISA reader at an excitation wavelength of 485 nm and emission wavelength of 535 nm. 

### 2.10. Apoptosis Analysis

After 24 h of IL-1β, IFN-γ, *Spirulina* extract, or PC exposure, 50,000 cells per well were seeded in 96-well culture plates (Greiner Bio-One Ltd., Stonehouse, UK), and caspase-3/7 activity was determined using the Apo-ONE^TM^ homogeneous caspase-3/7 assay (Promega) according to the manufacturer instructions, as an indicator of the degree of cell apoptosis. 

Cell apoptosis was also measured by detecting the extent of DNA fragmentation with a terminal deoxynucleotidyl transferase dUTP nick-end labeling (TUNEL) assay (Promega) in accordance with the manufacturer’s guidelines. In brief, treated RINm5F cells (1 × 10^5^ cells per well) were cultured on cover glass in 4-well plates. The cells were then fixed with 4% paraformaldehyde followed by incubation for 25 min at room temperature. The cells were washed with PBS, permeabilized with 0.2% Triton X-100 for 30 min, and 50 μL TUNEL reaction mixture was added followed by incubation for 60 min at 37 °C. The nuclei of cells were stained with DAPI (1:1000 in PBS) for 15 min, washed with PBS three times, and observed under laser-scanning confocal microscopy (Zeiss LSM 510).

### 2.11. Animal Model of Type 1 Diabetes

Specific pathogen-free male Wistar rats (5–6 weeks old) were obtained from Central Lab. Animal Inc. (Seoul, Korea). The rats were acclimated for one week before the experiment with free access to a standard commercial diet and housed under specific pathogen-free conditions at room temperature (23 ± 2 °C) with 50 ± 10% humidity and a 12-h light/dark cycle. This experiment was approved by the Eulji University Institutional Animal Care and Use Committee. To induce diabetes, the experimental animals were injected with STZ (Sigma) at a dose of 50 mg/kg body weight dissolved in citrate buffer (0.1 M, pH 4.5). The STZ-injected animals showed massive glycosuria and hyperglycemia within a few days, and diabetes was observed in 8 h-fasted rats at 72 h after STZ injection based on glucose concentration measurements (Accu-Chek, Roche, Basel, Switzerland), indicating that the model had been successfully established. Rats with a blood glucose level greater than 240 mg/dL were considered to exhibit diabetes and were used for the experiment. 

### 2.12. Animal Treatments

The animals were divided into six groups (with six rats per group) as follows: normal control group, water only; *Spirulina* extract group, administered *Spirulina* extract orally at 200 mg/kg body weight for 28 days; STZ-only groups assessed at 2 weeks and 4 weeks, respectively; and *Spirulina* + STZ groups assessed at 2 weeks and 4 weeks, respectively, in which *Spirulina* extract was administered orally at 200 mg/kg body weight for 2 or 4 weeks before diabetic induction.

**Figure 1:**
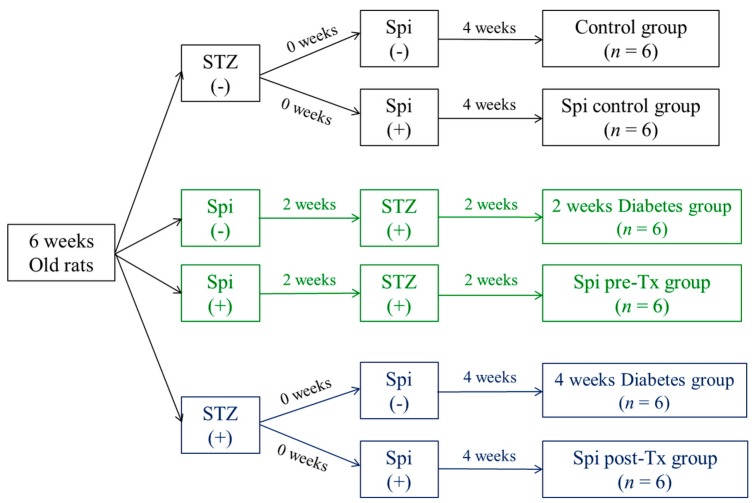
Schematic diagram of the in vivo experimental protocol.

Figure 1 presents the in vivo experimental scheme. At the end of the experiment (28 days), all animals were anesthetized and then sacrificed. Blood samples were collected in SST tubes (BD, Franklin Lakes, NJ, USA) and centrifuged at 3500 rpm for 5 min. The supernatants (serum) were stored at −80 °C before use.

### 2.13. Serological, Histological, and Immunohistochemistry Analysis

Serum insulin levels were measured using the ultra-sensitive rat insulin ELISA kit (Crystal Chem Inc., Downers Grove, IL, USA), and other serum-specific markers were estimated with the Lab Genomics Clinical Laboratories kit (LabGenomics, Seoul, Korea) according to the manufacturer instructions.

The pancreas tissue obtained from the experimental rats was collected, fixed in 4% paraformaldehyde, and processed for paraffin sectioning. Tissues were sectioned at a 4-μm thickness and stained with hematoxylin and eosin (H & E) to assess pathological changes of islet size under a light microscope with IMT i-solution software. Immunohistochemistry was performed with anti-insulin (1:200) and anti-nitrotyrosine (1:200) antibodies (Santa Cruz Biotechnology, Santa Cruz, CA, USA) and observed using the laser-scanning confocal microscope (Zeiss LSM 510).

### 2.14. Statistical Analysis 

The data are presented as means ± standard error of mean (SEM) of three independent experiments. Student’s *t*-test was used for comparisons between two experimental groups with Excel (Microsoft Corp, Redmond, WA, USA). Differences at *p* < 0.05 were considered statistically significant.

## 3. Results

### 3.1. Morphological Properties, Nutrients, and Protein Composition of S. maxima

**Figure A1:**
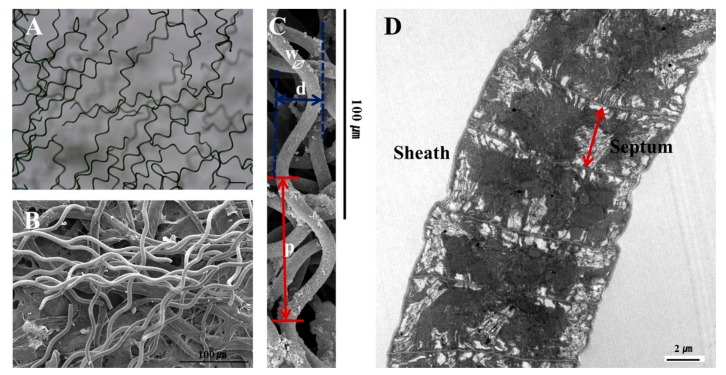
Morphology of *Spirulina maxima*. (**A**) Light micrograph. Original magnification, 100×; (**B**) Scanning electron micrograph. Original magnification, 350×; (**C**) The width (w) of the trichomes is 6–12 m, the pitch (p) is 12–72 m, and the diameter (d) is 30–70 m. Original magnification, 500×; (**D**) Longitudinal section along the trichome, illustrating the thin, diffluent, fibrillar, net-like sheath enveloping the trichome. The regularly spaced cross walls form a septum that divide the trichome into cells. Original magnification, 8600×.

Under light microscopy, *S. maxima* showed typical morphological features of filamentous cyanobacteria (Figure A1). Table 2 shows the nutrient composition of the *S. maxima* strain used in the experiments, including a high amount of proteins, minerals, vitamins, essential fatty acids, amino acids, and especially antioxidant proteins such as vitamin E and β-carotene.

**Figure A2:**
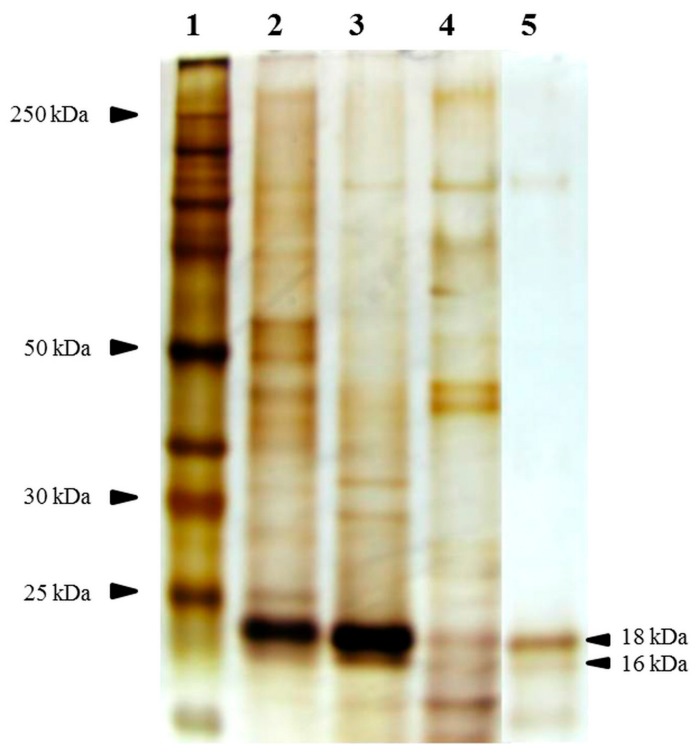
Protein profiles of *Spirulina* extracts determined by SDS-PAGE and silver nitrate staining. Lane 1, marker; Lane 2, *Spirulina* whole lysate; Lane 3, distilled water (DW) extract; Lane 4, ethanol (EtOH) extract; Lane 5, phycocyanin (Sigma, P-2172). Arrowheads indicate C-phycocyanin α, β-chain.

Based on 12% SDS-PAGE analysis, the protein profiles of DW extracts were more similar to those of the *Spirulina* whole lysate than to those of the EtOH extracts, and had a greater PC content (Figure A2).

### 3.2. Spirulina Extract Protects against IL-1β- and IFN-γ-Induced Cytotoxicity

**Figure 2:**
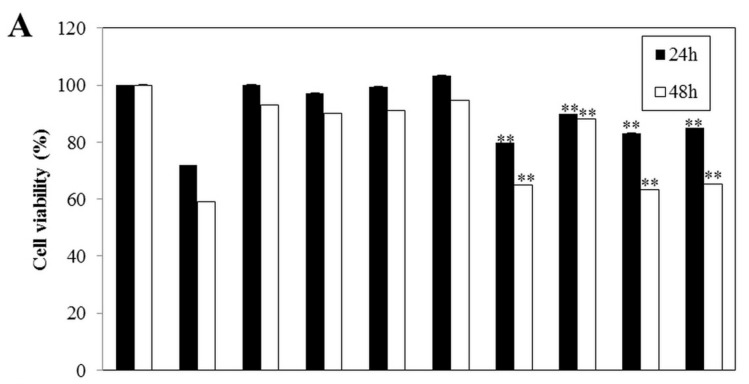

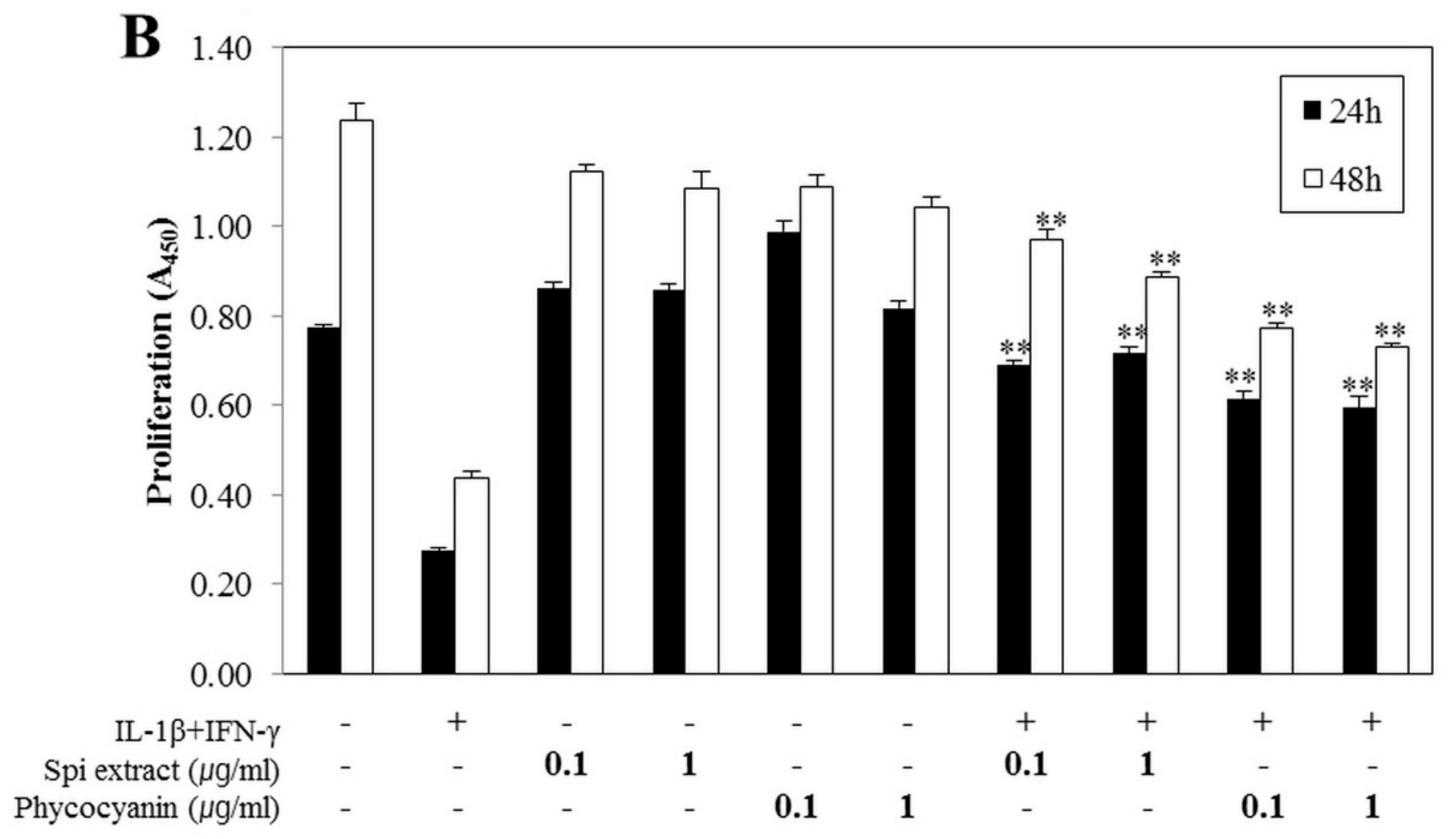
*Spirulina* extract protects against IL-1β- and IFN-γ-induced cell death. (**A**) Cell viability and (**B**) proliferation determined by the XTT assay and BrdU incorporation assay, respectively. The cells were co-treated with or without IL-1β (50 U/mL), IFN-γ (100 U/mL), and *Spirulina* (Spi) extract or PC (100 ng, 1 μg/mL) for 24 and 48 h. Each value represents the mean ± SEM of three independent experiments. The two-sample *t*-test was used to compare the results between the control group and treatment group. ** *p* < 0.01, significant difference between the Cytokine-treated and Cytokine + Spi extract or Cytokine + PC groups.

After treatment with IL-1β (50 U/mL) and IFN-γ (100 U/mL) for 24 h and 48 h, the cells showed an approximate 70% and 60% reduction in cell viability relative to control cells, respectively. Co-treatment with *Spirulina* extract or PC increased cell viability in a dose-dependent manner, thus attenuating the cytokine-induced cytotoxic effect (Figure 2A). At 0.1 and 1 μg, *Spirulina* extract increased the cell viability of cytokine-treated cells to approximately 80% and 90% in comparison with control cells, respectively, and there was no significant difference in cell viability between groups treated with *Spirulina* extract or PC and control cells. This protective effect of *Spirulina* extract on cytokine-induced growth inhibition was confirmed using the BrdU incorporation assay (Figure 2B). 

### 3.3. Spirulina Extract Inhibited NF-κB Activation and Downstream Gene Expression

**Figure 3:**
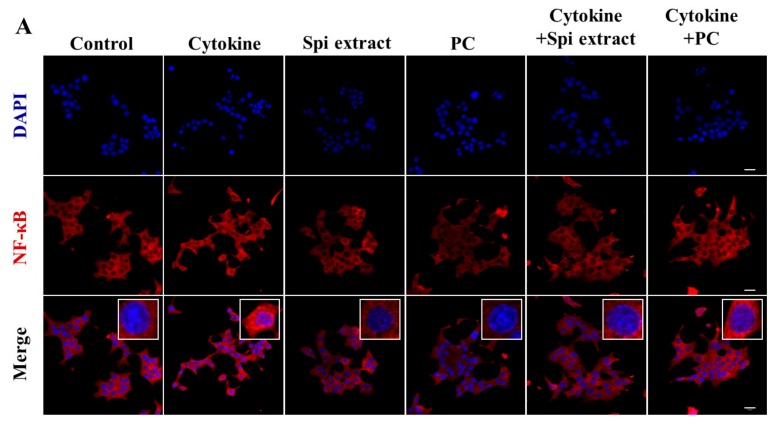

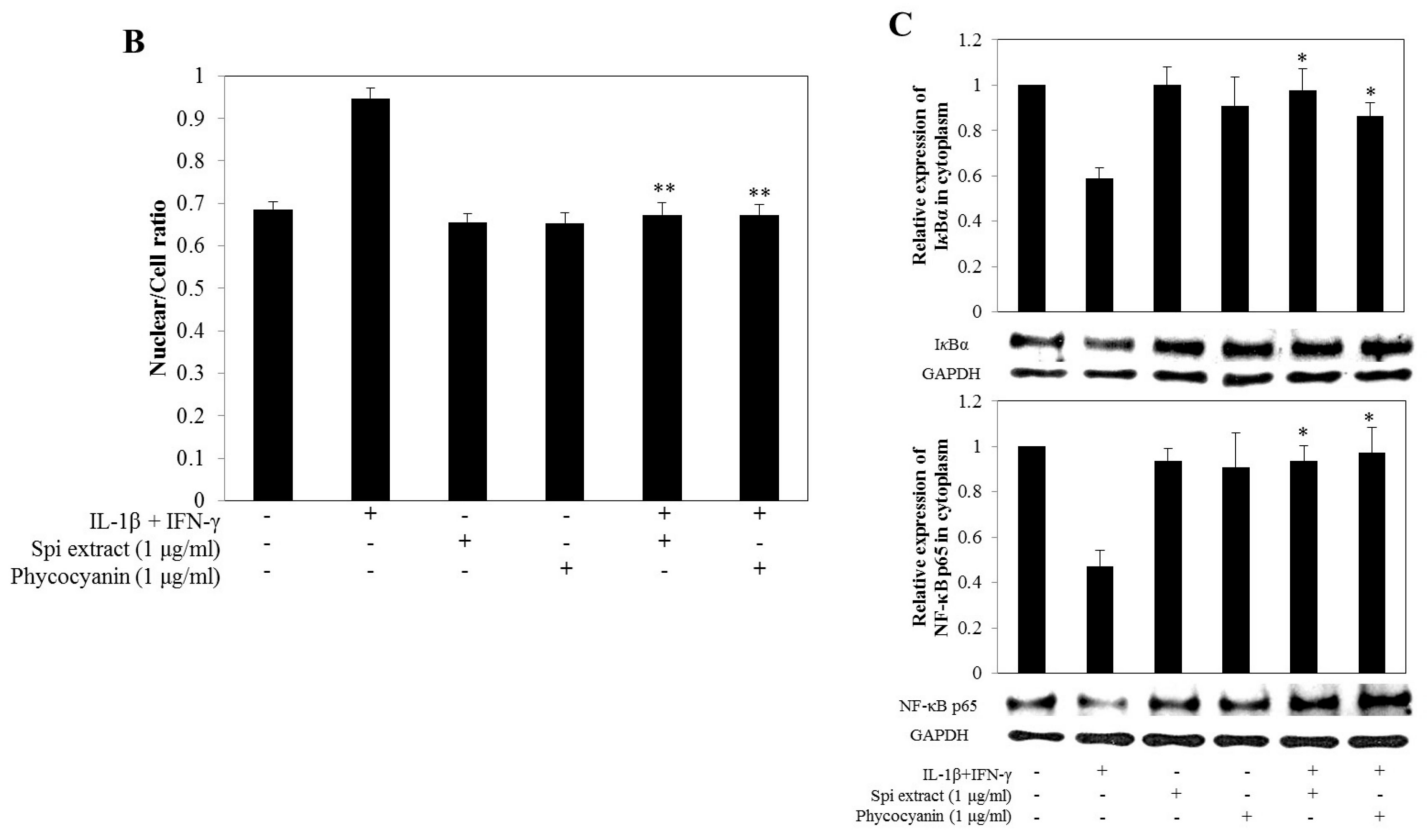
*Spirulina* extract inhibits IL-1β- and IFN-γ-induced NF-κB activity, translocation of p65 to the nucleus, and IκBα degradation. RINm5F cells were co-treated with *Spirulina* (Spi) extract or PC, and IL-1β and IFN-γ for 24 h. (**A**) NF-κB activity was observed by confocal microscopy (magnification, 400×); (**B**) The relative intensity of NF-κB activity was analyzed by confocal microscopy software; (**C**) Western blot analysis of IκB and NF-κB p65 in the cytoplasm fraction. The relative expression of proteins was determined using SYNGENE Gel Image software and the control band was given an arbitrary value of 1. Each value represents the mean ± SEM of three independent experiments. The two-sample *t*-test was used to compare the results between the control group and treatment group. * *p* < 0.05, ** *p* < 0.01, significant difference between the Cytokine-treated and the Cytokine + Spi extract or Cytokine + PC group.

Cytokine-treated cells showed increased translocation of NF-κB p65 (red) to the nucleus [8] compared to control cells (Figure 3A,B); this translocation was markedly inhibited following treatment with *Spirulina* extract or PC. Moreover, IκB**α** protein expression in the cytoplasm decreased because cytokine treatment increased the degradation of IκB**α** protein. NF-κB p65 protein expression in the cytoplasm decreased, as cytokine treatment increased the translocation of NF-κB p65 to the nucleus, which was decreased by *Spirulina* extract or PC (Figure 3C).

### 3.4. Spirulina Extract Modulates NF-κB-Dependent Gene Expression and ER Stress Marker Expression

**Figure 4:**
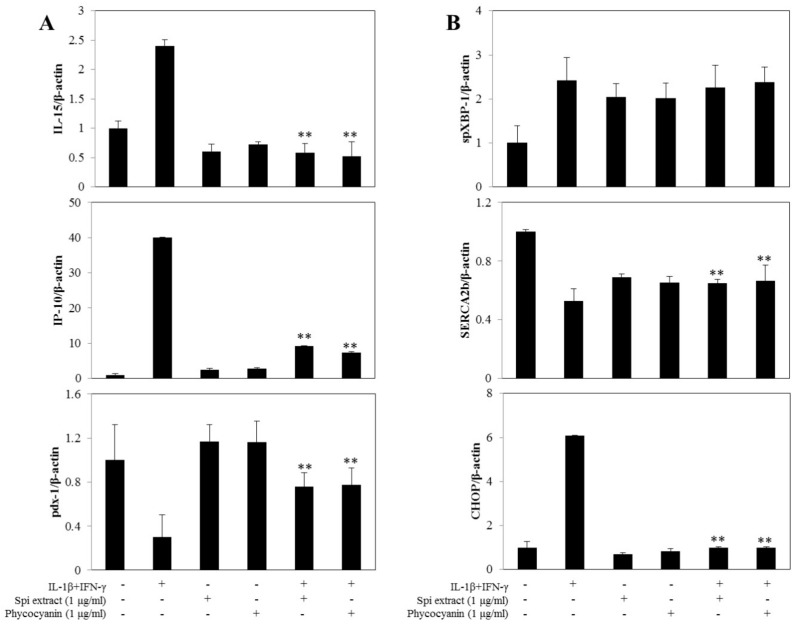
*Spirulina* extract modulates NF-κB-dependent and ER stress marker gene expression. RINm5F cells were co-treated with *Spirulina* (Spi) extract or PC, and IL-1β and IFN-γ for 24 h. (**A**) IL-15, IP-10, and pdx-1, and (**B**) spXBP-1 and SERCA2b mRNA expression levels were determined using real-time PCR; the housekeeping gene beta-actin was used to normalize the data. Each value represents the mean ± SEM of three independent experiments. The two-sample *t*-test was used to compare the results between the control group and treatment group. ** *p* < 0.01, significant difference between the Cytokine-treated and the Cytokine + Spi extract or Cytokine + PC groups.

The effects of *Spirulina* extract on NF-κB-dependent and ER stress marker gene expression were investigated by real-time RT-PCR. Cytokine-treated cells showed upregulated IL-15, IP-10, and CHOP expression compared with control cells, and these expression levels were significantly decreased by co-treatment with *Spirulina* extract or PC (Figure 4A,B). Cytokine treatment further downregulated SERCA2b expression, indicating the loss of differentiated β-cell functions by downregulation of pdx-1, ER calcium depletion, and ER stress; these effects were also reversed by co-treatment with *Spirulina* extract or PC (Figure 4A,B). These results indicate that *Spirulina* extracts or PC inhibited IL-1β- and IFN-γ-induced NF-κB activity and gene expression.

### 3.5. Spirulina Extract Attenuated Oxidative Stress

**Figure 5:**
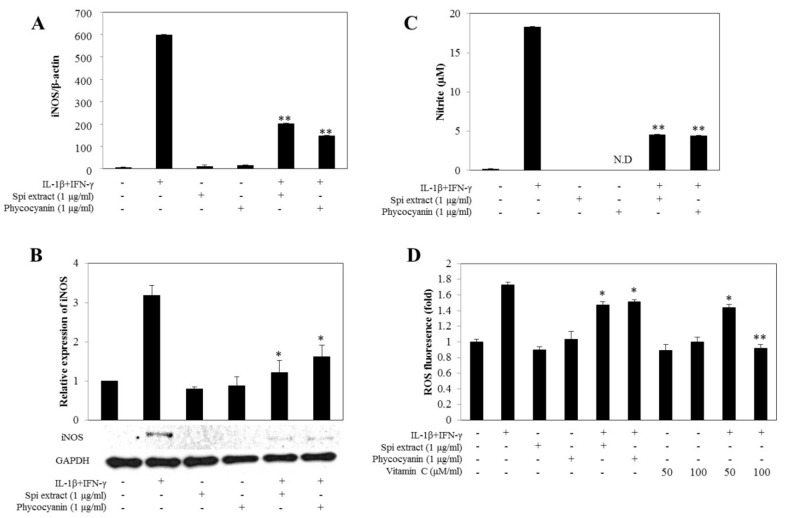
*Spirulina* extract inhibits cytokine-induced iNOS expression, NO production, and ROS generation. RINm5F cells were co-treated with *Spirulina* (Spi) extract or PC, and IL-1β and IFN-γ for 24 h. (**A**) The iNOS mRNA expression level was determined using real-time PCR, and the housekeeping gene beta-actin was used to normalize the data; (**B**) Western blot analysis of iNOS. The relative expression levels of proteins were determined using SYNGENE Gel Image software and the control band was given an arbitrary value of 1; (**C**) NO production assayed by the Greiss method; (**D**) ROS generation detected by the fluorescence intensity CH-H2DCFDA. Each value represents the mean ± SEM of three independent experiments. The two-sample *t*-test was used to compare the results between the control group and treatment group. * *p* < 0.05, ** *p* < 0.01, significant difference between the Cytokine-treated and the Cytokine + Spi extract or Cytokine + PC groups.

To determine whether *Spirulina* extracts regulated NO production by inhibiting iNOS expression, the mRNA and protein levels of iNOS were determined by real-time RT-PCR and western blot analysis, respectively. Cytokines increased the iNOS mRNA and protein expression levels of the cells, which were markedly inhibited with co-treatment of *Spirulina* extract or PC (Figure 5A,B). *Spirulina* extracts inhibited iNOS expression at both the mRNA and protein levels, which reduced NO expression. After treatment with cytokines in RINm5F cells for 24 h, production of nitrite (a stable oxidized product of NO) significantly increased. Treatment with *Spirulina* extract or PC significantly decreased this cytokine-mediated nitrite production (Figure 5C), which was well correlated with the reduced cytotoxicity (Figure 2). 

Moreover, treatment with *Spirulina* extract and PC decreased the cytokine-induced ROS production (Figure 5D). The experiment was repeated after 24 h incubation, because the gene expression level of iNOS was high at this point, which showed that *Spirulina* extract and PC decreased the formation of peroxynitrite. This further supports the inhibition of cytokine-mediated ROS production, as peroxynitrite would be prevented by decreased iNOS mRNA and protein expression (Figure 5A,B).

### 3.6. Spirulina Extract Protected against Cytokine-Induced Apoptosis through the MAPK Pathway

**Figure 6:**
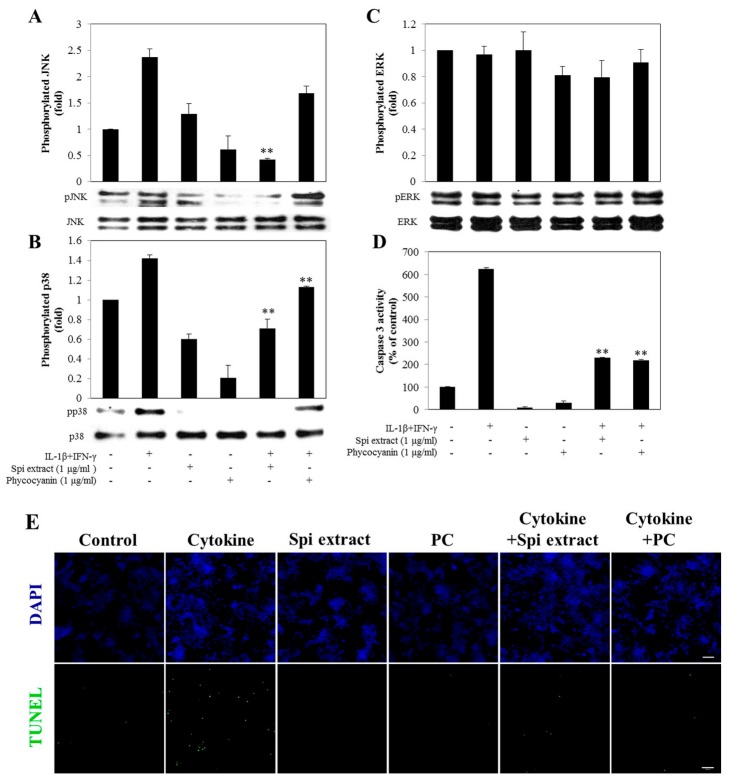
*Spirulina* extract attenuated RINm5F cells from cytokine-induced apoptosis. RINm5F cells were co-treated with *Spirulina* (Spi) extract or PC, and IL-1β and IFN-γ for 30 min. (**A**) Western blot analysis of JNK; (**B**) p38 phosphorylations; (**C**) ERK phosphorylations and (**D**) Caspase-3 activity in RINm5F cells. The relative expression levels of proteins were determined using SYNGENE Gel Image software and the control band was given an arbitrary value of 1; (**E**) DNA fragmentation detected by the TUNEL assay (magnification, 200×). TUNEL-positive nuclei due to DNA fragmentation appear as green areas. Each value represents the mean ± SEM of three independent experiments. The two-sample *t*-test was used to compare the results between the control group and treatment group. ** *p* < 0.01, significant difference between the Cytokine-treated and the Cytokine + Spi extract or Cytokine + PC groups.

Cytokine treatment resulted in the activation and phosphorylation of JNK and p38 in RINm5F cells (Figure 6A,B), whereas there was no difference in the level of phosphorylated ERK compared to control cells (Figure 6C). Treatment of RINm5F cells with *Spirulina* extract suppressed this cytokine-induced expression of phosphorylated JNK and p38 (Figure 6A,B). Taken together, these results demonstrate that *Spirulina* extract protects RINm5F cells from cytokine-induced cell death by inhibiting activation of JNK and p38. 

Caspase-3, a downstream molecule of MAPKs in the apoptosis pathways, was activated at 24 h treatment with cytokines. Treatment of RINm5F cells with *Spirulina* extract or PC blocked this activation of caspase-3 (Figure 6D). The TUNEL assay of cytokine-exposed RINm5F cells, which detects DNA fragmentation, confirmed this protective effect against cytokine-induced apoptosis. The number of TUNEL-positive cells significantly increased in cytokine-treated RINm5F cells, which was reduced after treatment with *Spirulina* extract or PC (Figure 6E).

### 3.7. Spirulina Extract Protects Islets from STZ-Induced β-Cell Destruction

**Figure 7:**
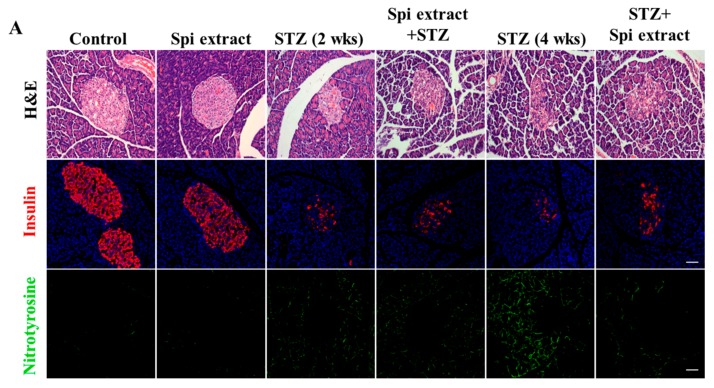

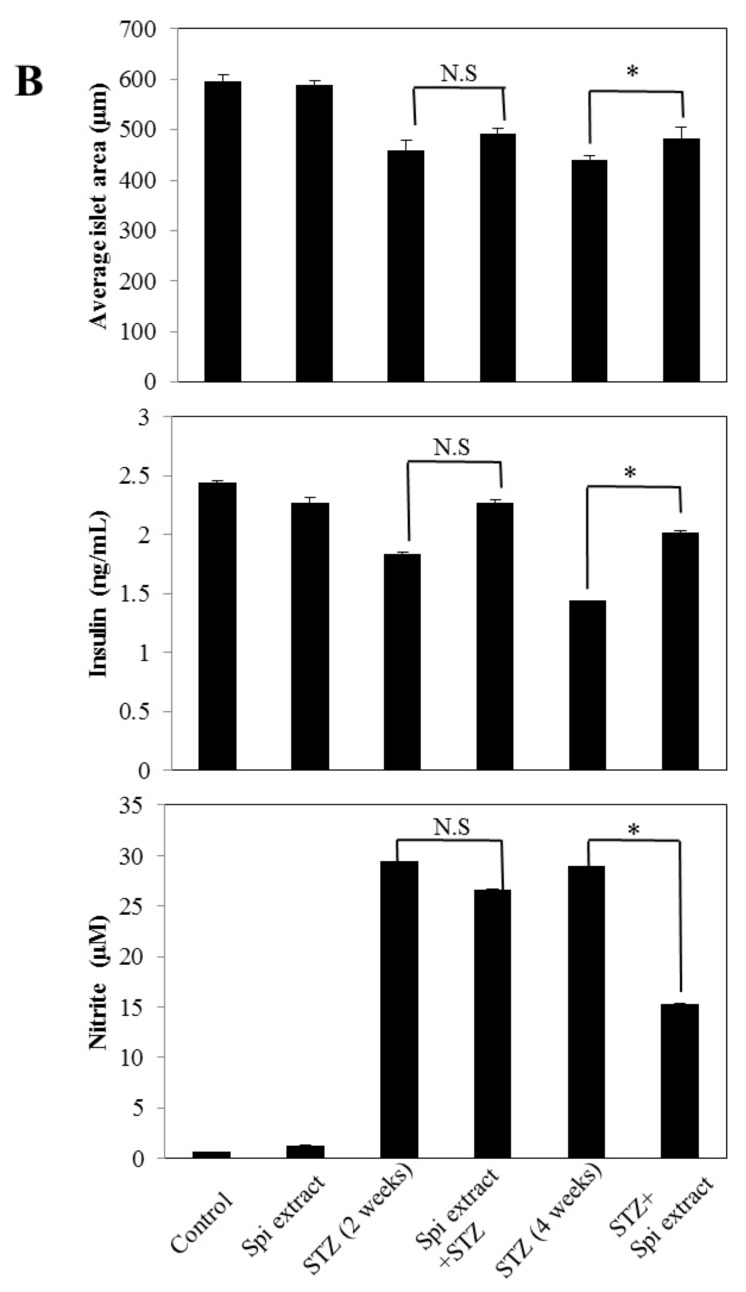
*Spirulina* extract protects islets from STZ-induced destruction. Type 1 diabetes was induced in Wistar rats through injection of STZ. (**A**) The cellular morphologies of islets were determined through staining with H&E. Islets were labeled with insulin antibody and nitrotyrosine antibody, and then examined by microscopy; (**B**) The size of islets was determined by IMT i-solution software. Levels of insulin and NO production in the serum were determined. Values are expressed as the mean ± SEM for six animals in each group. The two-sample *t*-test was used to compare the results between the control group and treatment group. N.S = Not significant. * *p* < 0.05, significant difference between the STZ-injected groups and the Spi extract + STZ or STZ + Spi extract groups.

The STZ-treated animals showed the typical pathophysiological feature of diabetes, including reduction of body weight, increase of blood glucose levels, and decrease of insulin levels. However, the blood glucose, total cholesterol, and triglyceride levels were significantly lowered and body weight loss was prevented in the groups treated with *Spirulina* extract in comparison with the control groups (Table 3). In addition, the levels of GOT and ALP, serum-specific markers that reflect liver status, were significantly increased in the STZ-treated groups, which were reduced with *Spirulina* extract administration. Moreover, H & E staining showed degenerative and necrotic changes of β-cells and a decrease in the islet size in the pancreas of the STZ-injected rats, which were normalized in the *Spirulina* extract groups (Table 4). In line with the serum enzyme results, immunohistochemical staining showed weak insulin reactivity and strong nitrotyrosine reactivity in the STZ-alone groups (Figure 7A,B), which recovered from ingestion of *Spirulina* extract.

## 4. Discussion

Several studies have shown that cytokine exposure increases ROS production to cause oxidative damage to β-cells [27]. This is the first study to demonstrate that *Spirulina* extract could reduce cytokine-induced apoptotic cell death and recover the cell damage from ROS production in RINm5F pancreatic β-cells. The mechanism of the cytotoxic effects of cytokines was determined to be through apoptosis induction by activation of caspase-3, which was related to oxidative stress from an imbalance in ROS production and antioxidant activity, resulting in activation of NF-κB, JNK, and p38 MAPKs to increase NO production. Indeed, NO has been shown to play a crucial role in regulating the cytokine-induced impairment of β-cell secretory function and cell death [36]. Co-treatment of *Spirulina* extract could protect β-cells against this cytokine-mediated cytotoxicity by reducing oxidative stress and cytokine-induced signaling pathways such as NF-κB, JNK, and p38. 

NF-κB is known to be an important factor in the pathological development of diabetes mellitus [41]. In particular, cytokine-induced NF-κB activation alters the gene expression profile of β-cells by affecting multiple genes involved in the β-cells differentiated state (pdx-1), ER Ca^2+^ homeostasis (SERCA2b), the attraction and activation of immune cells (IL-15, IP-10), ER stress (spXBP-1, CHOP), and the apoptosis of β-cells [16,42]. The inhibition of NF-κB activation or translocation leads to β-cell proliferation by suppressing IL-1β- and IFN-γ-induced β-cell damage or death [43,44]. Therefore, NF-κB is a crucial transcription factor in pancreatic β-cells. We showed that *Spirulina* extract suppressed NF-κB activation and nuclear translocation from IL-1β and IFN-γ exposure in RINm5F pancreatic β-cells, which is in line with the well-known anti-inflammatory effects of *Spirulina* and C-PC [45,46]. Table 2 shows the composition of *Spirulina*. Among the components listed, vitamins and amino acids are known to attenuate NF-κB signaling. Arginine reportedly causes IL-1β-mediated NF-κB activation in Caco-2 cells derived from human colon adenocarcinoma [47]. Moreover, cysteine, histidine, and glycine are known to inhibit NF-κB expression and IκBα degradation in human coronary arterial endothelial cells [48]. Vitamin K and E, which function as cofactors involved in various metabolic pathways, inhibit inflammatory cytokine-induced activation of NF-κB by repressing the phosphorylation of IKKa, which is upstream of NF-κB [49,50]. Furthermore, researchers have suggested that C-phycocyanin extracted from *Spirulina* also inhibits the ROS-Akt-NF-κB pathway [51,52]. Thus, *Spirulina* extract effectively degrades cytokine-induced IκB**α** to block the nuclear translocation of NF-κB p65 subunits and ultimately suppresses the downstream genes associated with β-cell apoptosis to protect the cells against NF-κB-mediated cytotoxicity. 

This oxidative stress induced by cytokines in β-cells plays an important role in the pathogenesis of type 1 diabetes. Pancreatic β-cells are considered to be particularly vulnerable to this toxicity because of the relative increase in ROS levels and decreased antioxidants [29,30]. The increased ROS stimulate NF-κB to activate downstream signaling pathways such as those leading to apoptosis [31,32] and MAPK [53]. Furthermore, reactions of superoxide radicals such as ROS and RNS promote β-cell death by forming peroxynitrite, another reactive species [54]. Given that *Spirulina* extract co-treatment suppressed the cytokine-mediated NO production that promotes iNOS, a downstream target of NF-κB, it can be used to attenuate oxidative stress and related pathways influenced by cytokines in β-cells. Thus, *Spirulina* extract has a cytoprotective effect against cytokines by suppressing ROS production.

These cytoprotective effects were confirmed in vivo, demonstrating a protective effect against the oxidative stress during the development of type 1 diabetes, as *Spirulina* extract protected against STZ-induced damage to the pancreatic islets of rats and NO production. 

MAPKs are well known to regulate various cellular processes, such as cell fate and differentiation, and are stimulated by growth or stress factors as well as oxidative stress [55]. Dysfunction of MAPK signaling has also been observed in diabetic patients [56], which may regulate the fates of pancreatic β-cells [57,58]. For example, activation of JNK and p38 kinase has been detected in pancreatic β-cells undergoing apoptosis [59,60]. In the present study, *Spirulina* extract attenuated the cytokine-induced activation of JNK and p38 kinase in RINm5F cells, further demonstrating a protective effect against oxidative stress. However, only *Spirulina* extract significantly decreased the activation of JNK, indicating a stronger effect on this kinase. 

Caspases are a family of cysteine proteases that are responsible for regulating apoptosis activated by various stimuli, and caspase-3 plays a key role in the cell death of mammalian cells [61]. In line with previous studies, cytokine treatment induced apoptosis via a caspase-3-mediated pathway in RINm5F cells, which was blocked by treatment with *Spirulina* extract. Therefore, *Spirulina* extract prevented cytokine-induced cytotoxicity in RINm5F cells mainly through inhibition of apoptosis.

In summary, we demonstrated that *Spirulina* extract protects pancreatic β-cells against cytokine-induced cell damage by inhibiting apoptosis and suppressing ROS production to attenuate oxidative stress both in vitro and in vivo. These findings confirm the antioxidant effects of *Spirulina* extract and highlight its potential for treating and preventing type 1 diabetes by enhancing the survival of β-cells under pathological conditions. 

## 5. Conclusions

The present study demonstrates that *Spirulina* extract inhibits cell apoptosis through cytokine-mediated cytotoxicity in RINm5F pancreatic β-cells. Activation of cytokine-mediated caspase-3, JNK, and p38 MAPK were attenuated by treatment with *Spirulina* extract. In addition, we identified that *Spirulina* extract possesses antioxidant activity, as indicated by the inhibition of intracellular ROS generation, especially NO generation. In cytokine-treated β-cells, NF-κB activation was attenuated and cell damage was inhibited by treatment with *Spirulina* extract. The in vivo study suggested that the ability of *Spirulina* extract to protect against the development of type 1 diabetes decreases blood glucose and NO levels, and morphological changes in the pancreatic islet. It seems likely that *Spirulina* extract suppressed oxidative stress in the type 1 diabetes model, thus indicating its important protective role in the disease. In conclusion, the antioxidant effect of *Spirulina* may be used to enhance the survival and reduce or delay cytokine-mediated β-cell destruction in type 1 diabetes.

## Figures and Tables

**Table 1:** Primers used to analyze mRNA expression by real-time PCR.

Accession No.	Gene	Sequence	Product Size (bp)	Annealing Temp (°C)	Cycles
NM.031144	β-actin	5′-CACCACAGCTGAGAGGGAAA-3′ 3′-TGGACAGTGAGGCCAGGATA-5′	472	66	40
NM.012611	iNOS	5′-AGGAGATGGTCCGCAAGGGA-3′ 3′-TCGTCGGCCAGCTCTTTCTG-5′	120	66	40
NM.022852	pdx-1	5′-GGTATAGCCAGCGAGATGCT-3′ 3′-TCAGTTGGGAGCCTGATTCT-5′	153	66	40
M57441	MCP-1	5′-CTTCTGGGCCTGTTGTTCA-3′ 3′-CCAGCCGACTCATTGGGATCA-5′	127	66	40
NM.013129	IL-15	5′-TGCAATGAACTGCTTTCTCCTGGAA-3′ 3′-GCTCCTCACATTCCTTGCAGCC-5′	158	66	40
NM.139089	IP-10	5′-TCGTTCTCTGCCTCGTGCTGC-3′ 3′-ATGGCCCTGGGTCTCAGCGT-5′	115	66	40
AF443192	spXBP-1	5′-CGCTTGGGAATGGACACGCT-3′ 3′-CTGCACCTGCTGCGGACTCA-5′	106	66	40
NM.001110823	SERCA2b	5′-CAAACAAACCGAGCCGGACG-3′ 3′-AACAACAGGCGTCATGGGGA-5′	122	66	40
U36994	CHOP	5′-TGCTGAAGAGAACGAGCGGC-3′ 3′-GGTGCAGACTGACCATGCGG-5′	104	66	40

**Table 2:** Compositions of *Spirulina maxima*.

General Composition	(%)	Amino Acid	(g/kg)
Moisture	3.2	Alanine	50.1
Protein	63.1	Arginine	30.9
Fat (Lipids)	7.2	Aspartic acid	59.4
Carbohydrate	22.1	Cystine	1.74
Minerals (Ash)	4.4	Glutamic acid	93.4
Fiber	13.2	Glycine	28.3
		Histidine	8.91
Vitamins	(mg/kg)	Isoleucine	23.2
β-carotene	1119	Leucine	46.7
Vitamin E	40	Lysine	24.0
Thiamin B-1	10	Methionine	10.1
Riboflavin B-2	10	Phenylalanine	23.2
Niacin B-3	144	Proline	19.9
Vitamin K-1	78	Serine	32.2
		Threonine	28.3
Essential fatty acids	(g/kg)	Tryptophan	5.9
Linoleic acid	13.0	Tyrosine	17.0
γ-linolenic acid	7.7	Valine	26.1

**Table 3:** Effects of streptozotocin (STZ) and *Spirulina* (Spi) extract on body weight; levels blood glucose, cholesterol, TG, BUN, creatinine.

	Initial Weight (g)	Final Weight (g)	Glucose (mg/dL)	Total Cholesterol (mg/dL)	TG (mg/dL)	BUN (mg/dL)	Creatinine (mg/dL)
Control	230.0 ± 5.32	346.6 ± 4.21	2.00.5 ± 15.3	81.0 ± 1.47	54 ± 6.9	20.6 ± 1.0	0.5 ± 0.01
Spi	235.0 ± 4.20	353.0 ± 3.65	252 ± 16.1	77.0 ± 1.05	48.0 ± 5.9	20.7 ± 0.4	0.6 ± 0.02
STZ (2 weeks)	235.0 ± 5.44	284.0 ± 7.55	522.3 ± 4.36	125.5 ± 3.24	414.0 ± 37.28	38.8 ± 2.05	0.5 ± 0.02
Spi + STZ	230.0 ± 6.03	295.3 ± 7.35	469.7 ± 19.93	108.0 ± 2.22	360.0 ± 14.71	31.1 ± 1.10	0.5 ± 0.02
STZ (4 weeks)	260.0 ± 2.56	234.8 ± 13.01	639 ± 7.8	129.3 ± 5.31	232 ± 34.2	43.7 ± 3.0	0.5 ± 0.02
STZ + Spi	260.0 ± 5.13	284.0 ± 6.97 ^a^	574 ± 16.7 ^a^	105.3 ± 1.83 ^a^	126.8 ± 11.1 ^a^	39.9 ± 2.0	0.5 ± 0.02

Values are expressed as means ± SEM for six animals in each group. ^a^ *p* < 0.05, significant difference between the STZ (4 weeks)-treated and the Spi extract-pretreated group (Spi + STZ).

**Table 4:** Measurement of liver function in the diabetic (STZ) and *Spirulina* (Spi) extract-treated serum.

	Total Protein (g/dL)	Albumin (g/dL)	GOT (IU/L)	GPT (IU/L)	ALP (U/L)	γ-GT (IU/L)
Control	6.0 ± 0.02	4.5 ± 0.03	151.8 ± 2.63	53.4 ± 3.07	955.4 ± 34.35	2.0 ± 0.00
Spi	6.2 ± 0.10	4.4 ± 0.04	121.7 ± 6.53	54.4 ± 2.20	856.8 ± 31.42	2.0 ± 0.40
STZ (2 weeks)	5.6 ± 0.05	3.9 ± 0.04	325.0 ± 17.68	163.7 ± 2.62	3422.0 ± 77.89	2.0 ± 0.00
Spi + STZ	5.6 ± 0.05	3.9 ± 0.05	212.7 ± 8.37 ^a^	166.3 ± 15.11	2159.3 ± 223.77 ^a^	2.0 ± 0.00
STZ (4 weeks)	5.4 ± 0.05	3.7 ± 0.06	367.7 ± 1.31	358.0 ± 44.25	3725.6 ± 154.05	11.3 ± 1.43
STZ + Spi	5.4 ± 0.05	3.6 ± 0.03	232.0 ± 23.90 ^b^	208.7 ± 7.96	2798.0 ± 140.70 ^b^	2.3 ± 0.20 ^b^

Values are expressed as means ± SEM for six animals in each group. ^a^ *p* < 0.01, significant difference between the STZ (2 weeks)-treated and the Spi extract-pretreated group (Spi + STZ). ^b^ *p* < 0.01, significant difference between the STZ (4 weeks)-treated and the Spi extract-post treated group (STZ + Spi).
